# Fitness Use Innovativeness, Usage Patterns and Revisit Intention: The Moderating Role of Other-Efficacy

**DOI:** 10.3390/bs13040307

**Published:** 2023-04-04

**Authors:** Theeralak Satjawathee, Stephen Donald Strombeck, Shih-Tung Shu, Ching-Hung Chang

**Affiliations:** 1 International College for Interdisciplinary Studies, Payap University, Super-Highway Chiang Mai—Lumpang Road, Amphur Muang, Chiang Mai 50000, Thailand; 2 The School of Business, Rocklin Campus, William Jessup University, 2121 University Avenue, Rocklin, CA 95765, USA; 3 Department of Kinesiology, Health, and Leisure Studies, National University of Kaohsiung, 700 Kaohsiung University Rd., Nanzih District, Kaohsiung 811, Taiwan

**Keywords:** fitness use innovativeness, other-efficacy, usage patterns

## Abstract

Extant research has confirmed the importance of consumer innovativeness toward innovation adoption, but relatively little is known about the relationship between fitness use innovativeness, post-adoption behavior and the moderating role of fitness consumer’s efficacy belief. This study aims to examine the moderating role of other-efficacy on the relationships between the fitness player’s usage patterns (usage variety and usage frequency) under the influence of use innovativeness, and revisit intention within the context of fitness services. This study utilizes the diffusion model for conceptual development. The proposed hypotheses are empirically tested using fitness players from a public sports center. There were 205 valid questionnaires obtained for quantitative data analysis. The findings confirm that the fitness player’s use innovativeness has a direct impact on usage variety and usage frequency, while the player’s training partner efficacy positively moderates the usage patterns and revisit intention. Based on the extent of fitness use innovativeness and training partner efficacy, we categorize fitness customers into four segments. The managerial implications for each segment are then discussed.

## 1. Introduction

Service business, such as theme parks and themed restaurants, often segment consumers in lieu of innovative behaviors to identify new segments [1]. This is also true within the “facility-driven” health club industry [2]. With more than 200,000 health clubs worldwide and 162 million members, this highly competitive industry is valued at approximately USD 83.1 billion [3]. To effectively compete in this industry, chain health clubs have pursued a wide range of fitness services to attract different customer segments, which exhibit high variety seeking behaviors [4]. As such, these service firms must frequently reinvent their services to meet consumers’ novelty needs and explore better approaches to satisfy customers’ expectations [4].

Many fitness consumers are high-frequency users [5]. The perceived novelty of fitness services though is strongly impacted by such variants as visit frequency and duration of stay. Hence, a customer’s level of satisfaction with a fitness service is likely to change once they are familiar with the facilities or equipment. In a highly facility-driven service setting such as a fitness center, customers active usage of new innovations is imperative for success [6]. Specifically, fitness center managers need to understand what factors influence customer tendencies toward use innovativeness within the fitness context.

Use innovativeness is defined as using an existing product or service in a new way [7]. A user adopting a new and creative approach to using a product or service may even lead to a unique usage pattern [7]. Through this process, the use of a previously adopted product can resolve novel consumption problems [8]. Use innovativeness leads to variety-seeking behavior in the usage context. Clearly, consumers with high use innovativeness tend to develop high creativity and to attempt diverse applications of a product [9,10].

Innate consumer innovativeness is a generalized unobservable trait [8,11] and predisposition toward the willingness to try new things [12]. Many studies have proclaimed that consumer innovativeness affects the adoption rate of new innovations [10,13] in the domain of high-tech consumer products and technology-based self-services. Some others have regarded consumer innovativeness as an important determinant of attitude in the technology acceptance model [14,15]. Although these studies have investigated the relationship between consumer innovativeness and pre-adoption behavior within the context of technology-based products, little is known about the effect of use innovativeness on post-adoption behavior in a services consumption setting [8]. As a consumer’s innate innovativeness provides the drive to adopt innovations for both hedonic and utilitarian reasons [16,17], this innate need for variety should lead services providers to consider offering new services, which refresh consumer experiences and enhance the service firm’s market performance [18].

Other-efficacy (OE) belief, an individual’s confidence in another person’s ability to perform particular behaviors [18], is also called proxy control. It is a socially mediated form of perceived control that involves the relinquishment of some personal control to an intermediary party in order to achieve specific desired outcomes [19]. Social cognitive theory suggests that an individual’s perception of the capabilities of partners or trainers affect their own performances [20,21]. Individuals resort to proxy control when they have not developed adequate means to reach their desired outcomes. They believe a third party can better help them achieve desired outcomes, or do not want the personal responsibility of direct control [20]. The present study investigates the concept of other-efficacy in the context of fitness customers, which refers to the level of confidence that an individual has in the ability and knowledge of their training partner or instructor to perform various exercises and operate fitness equipment. Other-efficacy is a subjective perception that can vary across individuals, with some perceiving their partner or trainer as highly competent, while others may view them as less capable. For example, a training partner who may not possess the necessary knowledge to operate the equipment can still be perceived as having some level of efficacy by the fitness player.

Other-efficacy is essential for a fitness center. However, their importance can vary depending on the business model of the fitness center. For instance, low-cost fitness centers offer affordable and accessible fitness facilities with basic equipment, minimum staff, and low overhead costs [22]. They typically charge lower membership fees and aim to attract a larger number of customers by offering basic services such as free weights, cardio machines, and group fitness classes. As a result, these fitness centers tend to have a high volume of members, and their primary source of revenue is from membership fees [23]. Examples of low-cost fitness center chains include Planet Fitness, Crunch Fitness, and 24 Hour Fitness. On the other hand, premium fitness centers focus on providing a high-quality fitness experience for their members by offering state-of-the-art equipment, personalized training programs, and a variety of amenities such as spa services, pools, and nutritional counseling [24]. These fitness centers charge higher membership fees and aim to attract a smaller number of members who are willing to pay a premium for an exceptional fitness experience. As a result, their primary source of revenue is from membership fees, but they may also generate additional revenue from selling supplements or personal training sessions. Examples of premium fitness center chains include Equinox, Life Time Fitness, and Gold’s Gym.

Recent investigations have investigated the relationships between OE beliefs and behavioral adaptation across a variety of domains [25]. For example, patients are more likely to adhere to health behavior change if they have a greater perceived competence in their health care provider, and believe their provider can effectively influence their personal health outcomes [26]. Another medical study showed that patients’ beliefs in their doctors’ capabilities supplemented their own OE belief for managing symptoms and overcoming serious illness [27]. These findings highlight the salient role of OE in health-related behaviors that involve social interactions with a perceived credible source (e.g., rehabilitation therapist).

Other studies [19,28] have highlighted relationships between other-efficacy and important correlates of exercise behavior, such as exercise self-efficacy, exercise adherence, intention to exercise, and exercise frequency. Notably, they treated other-efficacy primarily as an antecedent to its correlational behavioral adoption in nature, while very few studies have focused on the moderating role of other-efficacy.

Although Yim et al. [29] examined the synergistic effects of self-efficacy and other-efficacy, including congruence and incongruence levels of the efficacy beliefs, the influence of other-efficacy on fitness customer’s post-behaviors remains to be more thoroughly investigated. Examining this relationship is important because the fitness service environment provides a multiplicity of social interactions, wherein customers interpret and reflect on their own performance accomplishments relative to the performances of others while receiving evaluative feedback from partners and instructors. Such an investigation may enrich our insights in segmenting fitness customers, developing marketing programs around new uses, and fulfilling customer novelty-seeking needs.

The purpose of this study is to explore fitness customers’ usage patterns in the midst of the intervention of other-efficacy for the highly facility-driven service environment. Specifically, it intends to examine the moderating effect of a fitness customer’s other-efficacy on the relationships between usage patterns and reusage intent. Furthermore, this research distinguishes efficacy about one’s partner (other-efficacy) to consider it from the perspectives of both the customer and his/her partner or trainer. This effort extends the examination of efficacy from a single-party perspective to a multiparty, interdependent relationship perspective. Finally, this study suggests an intriguing implication to formulate effective marketing strategies for practitioners (e.g., marketers of fitness centers) and should shed new light on unexplored but critical impacts of other-efficacy and directions for future studies.

## 2. Conceptual Development

### 2.1. Theoretical Foundation

In the theoretical foundation of diffusion models, two types of models have been proposed to explain the relationship between individual innovativeness and usage pattern. According to Rogers [30], innovativeness refers to the different reactions of an individual to a new idea, practice, or object due to differences in their innovativeness. The first model is the adoption-diffusion (AD) model, which focuses on the rate and time of adoption. The second model is the use-diffusion (UD) model, which examines the rate of use and variety of use [9].

Research has shown that inherent novelty seeking, which is a personal trait, can affect one’s usage variety [7,8,9]. Ridgway and Price [7] proposed the concept of innate innovative, which consists of adaptive, vicarious, and use innovativeness as antecedent variables for actualized use innovativeness. Innate use innovativeness is defined as the attraction and creativity in using products in new ways, where the introduction and production of new product use, not the adoption of new products, is the focus.

### 2.2. Use Innovativeness and Usage Patterns (Usage Frequency and Usage Variety)

Use innovativeness may affect usage patterns. Frequently, usage patterns are conceptualized on two distinct dimensions: usage frequency and usage variety [8,9]. Usage frequency (UF) refers to the time of product or service, and usage variety (UV) is the ways in which a product or service is used. For example, in the context of health club customers, UF is the number of times they visit health clubs in a given period (i.e., week, month, or year), and UV is the different ways and frequency the product is used [8]. Equipment of a typical health center could include cardio machines (elliptical cross trainers, sitting bike, upright cycle, treadmill, spinning bike, etc.) and weight training equipment (e.g., horseback riding, climber, leg press, pec-deck machine, PEC machine/butterfly, shoulder press, lower back, chest press, etc.). As UI is viewed as encompassing both attraction or openness to new product uses and creative ability, consumers with high UI may seek opportunities to use products in new ways rather than waiting to respond to a consumption situation. Studies have evidenced that a greater use innovativeness results in a higher usage variety [8,9]. Shih and Venkatesh [9] found a positive relationship between use innovativeness and usage variety, but not on usage frequency in a context of technology use diffusion. However, one other study [8] found that UI positively affects UF and UV in the context of home electronics use (e.g., VCR, microwave oven, and food processor). Chang et al. [5] found that fitness consumers’ adoption innovativeness positively influences their re-visit frequency and duration of stay. As such, this study proposes the following two hypotheses:

**Hypothesis** **1 (H1).** *Use innovativeness positively affects usage variety.*

**Hypothesis** **2 (H2).** *Use innovativeness positively affects usage frequency.*

### 2.3. Usage Patterns (Usage Frequency and Usage Variety) and Revisit Intention

Post-adoption usage behavior has been validated as a predictor of purchase intention for the subsequent innovation [31]. This study found that a high usage rate of basic and innovative functions of successive versions of innovations, like cell phones, may lead to strong purchase intentions for the next-generation product. Specifically, usage of the innovative functions has a significantly stronger impact on the purchase intention than simple usage of the basic functions. Cell-phone users blending the technology successfully into their lives may probably be the least resistant to acquiring similar but improved technologies. Additionally, past successful use experiences might reduce the level of perceived risks involved while heightening the expectation of possible benefits [9,32]. Hahn et al. [33] refer to positive effects of “direct product experience” as a key precondition for innovative adopters to become repeaters. When people gain greater technology mastery from frequent usage, they often develop more efficiency and confidence for their continued use of the technology and intention to repurchase in the future.

In the use-diffusion (UD) context, intense users, who score high on both usage variety and usage rate, may be considered as use innovators par excellence [9]. This study demonstrated that users who exhibited an intense UD pattern are more inclined to be satisfied with the technology than the users who exhibited limited use. Son and Han [34] confirmed that high innovative use and frequent use of technology-based products tends to produce higher retention levels, because new ways of using these products have been learned. Satjawathee et al. [35] also found that a higher level of usage variety and frequency lead to a higher satisfaction and revisit intention on fitness services.

Sometimes, repeated use of a product induces boredom for the buyer [36] because use of the product is no longer novel or complex [37]. In contrast, alternating usage of familiar items in a product category can raise the level of stimulation for consumers [38]. Although little empirical evidence has purported the relationship between usage patterns and fitness consumers’ revisit intention, this study argues that both usage variety and usage frequency, in lieu of use innovativeness, may have a positive impact on a fitness customer’s re-patronage intention. That is, the innate needs of variety-seeking may arouse fitness customers to obtain stimulation in their workout routines by alternating between familiar equipment simply for a change of pace. In other words, customers with higher variety-seeking needs may infuse their body building and workout procedures with new routines to achieve a satisfactory level of stimulation [37,39]. Along with the previous hypotheses, we expect to find support for the following hypotheses:

**Hypothesis** **3 (H3).** *Usage variety has a positive effect on revisit intention.*

**Hypothesis** **4 (H4).** *Usage frequency has a positive effect on revisit intention.*

### 2.4. Other-Efficacy and Its Moderating Effect on Usage Pattern (Usage Frequency and Usage Variety)

Fitness customers’ other-efficacy from partners or trainers may boost their confidence in utilizing the fitness equipment innovatively and multifariously. One prior study argues that professional instructions from significant others may lead to a higher fitness involvement and an increase in usage rates [8]. Namely, beliefs about other-efficacy at providing personally-desired responses may affect one’s sense of satisfaction with, and intention to persist in the relationship, despite conflict or other difficulties [19]. Therefore, fitness customers with higher other-efficacy can raise their own efficacy and involvement, increase the usage rate and the dexterity of utilizing the fitness devices, and produce higher revisit intentions than those with lower levels of other-efficacy.

Similarly, some customers may lack the skills or knowledge needed to develop a strong sense of personal efficacy for exercise, and may thus resist using unfamiliar fitness equipment. Under such conditions, these customers might have more confidence in those trainers with skillful resources [19]. Professional assistance from trainers and knowledgeable partners may enable those consumers who have low efficacy to gain confidence when they face challenges of new uses. Thus, favorable appraisals of their partners and trainers efficacy are likely to strengthen the usage behavior and lead to a strong sense of a collaboration alliance, great commitment to, and truthful acceptance of the instructor’s feedback based on a belief in their capabilities. In other words, comfortability with the help and advice of a trainer will likely lead them to try using varied functions of the equipment and developing adequate means to achieve desired outcomes. Thus, this study argues that innovative consumers are prone to use fitness equipment in more various ways and to increase usage rate if they perceive partners and trainers to be highly capable. As such, the effect of usage variety and usage frequency on fitness customer loyalty, in terms of revisit or reuse of the facilities, would be strengthened if the customers have a higher other-efficacy belief in their partners or trainers. This reasoning suggests the following two moderating hypotheses:

**Hypothesis** **5 (H5).** *Other-efficacy moderates the effect of usage variety on revisit intention.*

**Hypothesis** **6 (H6).** *Other-efficacy moderates the effect of usage frequency on revisit intention.*

## 3. Research Methodology

### 3.1. Participants and Data Collection

Taipei Nangang Sports Center (Taipei, Taiwan) was chosen as an appropriate context for the following reasons; although, it only represents one of the public sport centers. Their business models are all identical as they have to follow a standard BOT (build-operation-transfer) procedure for the general public.

Firstly, Taipei Nangang Sports Center is one of the largest public sports centers in Taipei, so it has a high flow of fitness-oriented people. Its fitness center is able to accommodate over one hundred customers and it possesses more than thirty varieties of equipment. Secondly, due to its convenient location, this community-based sports center attracts not only nearby residents but also commuters who work and/or study in the Nangang District, Taipei, Taiwan. Thirdly, this fitness center uses a pay-per-visit payment system, which measures the customer’s repatronage rate. Last, but not least, this fitness center offers an element of proxy control by providing an opportunity to examine customers’ other efficacy on their significant training partners or instructors. Specifically, customers and fitness instructors invest a considerable amount of time, effort, and resources to cultivate close relationships, which are both salient and relevant to this study [19].

The researchers of this study adopted the natural field settings methodology, which is widely recognized as an emerging approach for examining and collecting data on customers’ behavior [40]. Questionnaires were distributed, both during the week and on the weekend, to achieve a more representative sample. A total of 348 fitness customers were invited to complete the questionnaire while they finished their workout routines. A filter question (e.g., I often workout with some partners or trainer in this fitness center.) was designed at the end of this questionnaire. If the respondent selected “No” on this question, his/her questionnaire was excluded from the usable questionnaires. After removing the unusable questionnaires, a sample of 205 complete and usable responses remained for empirical analysis.

The sample for this study was comprised of 66.8% males and 33.2% females. Most respondents in the sample were under the age of 30, with 32.7% between 18 and 20 years old, and 43.4% between 21 and 30 years old. The level of education of the respondents was predominantly at the college or university level (63.9%). In terms of the duration of usage, most respondents (65.9%) spent 1 to 2 h at the fitness center for their workout routine. Regarding the frequency of usage, most subjects used the facility once (39%) or twice (32.2%) a week.

### 3.2. Measures

A fitness customer’s use innovativeness is defined as a tendency to use products or services in new and creative ways [7]. The measure used in this study is an edited version of use innovativeness scale that has been employed in previous research [9]. In the initial pilot test, two items demonstrated low validity scores and were crossed out. As a result, three items were selected to assess the facility-driven service environment. These items were carefully chosen based on their ability to effectively capture the intended construct. The items selected for this purpose were (1) “I am creative with fitness equipment”, (2) “I am curious about how fitness equipment works”, and (3) “I use fitness equipment in more ways than most people do”. The inclusion of these items ensured that the construct was measured accurately and comprehensively, thereby enhancing the reliability and validity of the study’s results.

Usage variety refers to the different applications for which a product is used and the different situations in which a product or service is used, regardless of how frequently the product or the service is used [8]. This construct was a measure of the twenty most commonly-used fitness equipment used by the respondents. Equipment includes five cardio machines (elliptical cross trainers, sitting bike, upright cycle, treadmill, and spinning bike) and fifteen weight training equipment (e.g., horseback riding, climber, leg press, pec-deck machine, PEC machine/butterfly, shoulder press, lower back, chest press, lat-machine with mid row, dumbbell, bench press, dip chin ab, seated leg curl, web-board, and row machine).

Usage frequency relates to how often the equipment is used (times of usage), regardless of the different applications for which the equipment is used [8]. Usage frequency was calculated in this study by multiplying the weekly frequency of visits and the duration of stay (in hours) for each visit. It is worth noting that both usage variety and usage frequency are considered to be behavioral measures, as they are derived from observed patterns of behavior rather than self-reported perceptions or attitudes. This approach to measurement allowed for a more objective and accurate assessment of the participants’ actual engagement with fitness services, which is critical for developing effective strategies to promote the adoption and continued use of such services.

For this study, we adopted the other-efficacy scale from Riggs et al.’s [40] personal efficacy belief scale. It was amended with a four-item scale to measure a fitness customer’s beliefs in the ability and confidence of their partners or trainers to assist him/her in using the fitness equipment. The scale used in this study includes the following items: (1) “I have confidence in my training partners’ ability to receive instruction on the usage of fitness equipment”, (2) “My training partners have excellent skills and ability to provide instruction on the usage of fitness equipment”, and (3) “I am proud of my training partners’ skills and ability to provide instruction on the usage of fitness equipment”. These items were selected based on their ability to effectively capture the participants’ beliefs regarding the efficacy of their training partners in providing instruction on fitness equipment usage.

Revisit intention measures the tendency of shoppers to repeatedly purchase goods or services at the same shop over time [41]. This construct was measured using the scale adopted from Zeithaml et al. [41] to assess continual usage intention of the fitness facility’s equipment. The items for this scale were (1) I would like to revisit this fitness center in the near future; (2) I will continue to use fitness equipment in this fitness center; (3) This fitness center would be my first choice over other fitness centers.

In order to accurately capture participants’ responses to the measuring items for each construct, a 7-point Likert scale was utilized in this study. Participants were asked to rate their level of agreement with each statement on a scale ranging from 1 (“strongly disagree”) to 7 (“strongly agree”). The use of this scale allowed for a more nuanced and detailed understanding of participants’ attitudes and beliefs regarding fitness service adoption and post-adoption behavior. Prior to administering the questionnaires, experts in the field of fitness research and fitness participants were consulted to ensure the clarity and comprehensibility of the measuring items. This process helped to ensure that the questions were accurately capturing the intended constructs, and that participants were able to provide meaningful and accurate responses.

### 3.3. Data Analysis

Structural equation modeling was used to validate the framework and test the hypothesized relationships using the Partial Least Squares (PLS) procedures with Smart-PLS 2.0 [42]. The variance-based PLS procedure was used because this distribution-free regression analysis technique is robust for small sample sizes and deviations from normality [43]. PLS is an appropriate structural equation modeling technique for studies that have not yet gained full recognition in the literature [42]. It is also good at mediation effect analysis and model examination since it is originally based on the concept of regression and path analysis. Both the measurement and structural models were assessed simultaneously. First, the validity and reliability of the measurement model were assessed, followed by the test of the structural model concerning the value of path coefficient (*β* value) and *R^2^* value [44].

## 4. Findings

### 4.1. Respondent Profile

In this study, a total of 205 participants were included, comprising of 66.8% males and 33.2% females. The majority of respondents fell within the age range of 18–20 years (32.7%), and 21–30 years (43.4%), indicating a preference for fitness services among the younger generation under 30. In terms of education, 63.9% of the participants had completed a college- or university-level education. In relation to the frequency of visits, the majority of respondents (65.9%) reported spending 1–2 h during their workout routine at the fitness center, with 71% of them exercising weekly, and 39% and 32.2% working out once and twice a week, respectively.

### 4.2. Measurement Model

The measurement model was assessed using means, standard deviations, and standardized loadings of each measuring item, as presented in Table 1. The cross-factor loadings of each corresponding construct ranged from 0.68 to 0.96, indicating a significant level of *p* < 0.001, which demonstrates good discriminant validity [45]. Furthermore, the convergent validity was found to be above the threshold with criteria, as indicated in Table 2. The Cronbach’s α scores of the five constructs exceeded 0.60 [46], and all values of composite reliabilities (CR) exceeded 0.70 [47], suggesting a high internal consistency of indicators measuring each construct, and thus confirming construct reliability. These findings indicate the robustness of the measurement model used in this study.

The average variance extracted (AVE) for each construct was significantly above 0.50 [48], proving a high internal consistency of each construct and thus confirming construct reliability. Furthermore, Table 2 shows that the coefficients range between 0.20 and 0.47. The square root of AVE for each construct was higher than corresponding inter-construct correlations. On this basis, the discriminant validity was also acceptable [48].

### 4.3. Structural Model

**Figure 1:**
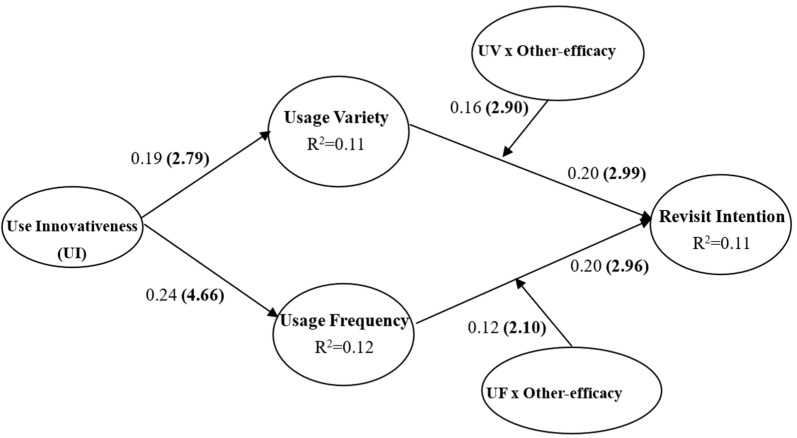
Path coefficients. The numbers in parentheses are *t*-values.

The hypothesized relationships were tested using the standard bootstrapping procedure with individual sign changes and the resample of three times of subjects [45] to generate t-values. As exhibited in Table 3 and Figure 1, the results of testing H1 and H2 indicate that use innovativeness has a significant effect on usage variety (β = 0.19, *t* = 2.79) and usage frequency (β = 0.24, *t* = 4.66), respectively. On this basis, H1 and H2 were supported.

H3 and H4 proposed that usage pattern, respectively, has a positive effect on revisit intention. As shown in Table 3 and Figure 1, the path coefficient (β = 0.20, *t* = 2.99) between usage variety and revisit intention is significant at *p* <0.01. Thus, Hypothesis 3 is supported. Additionally, the result of testing H4 indicated that the effect of usage frequency on revisit intention (β = 0.20, *t* = 2.96) is positively significant, lending support to H3. Thus, the empirical analysis manifests that usage patterns have a significant effect on revisit intention.

H5 and H6 posit that other-efficacy moderates both the relationships between use innovativeness and usage variety, and between use innovativeness and usage frequency. The interaction term was employed to test the moderating effect of other-efficacy in explaining the relationship between use patterns and revisit intentions. The effect of the interaction term (UV × Other-efficacy) on usage variety (β = 0.16, *t* = 2.90) achieved a significant level of *p* < 0.01, thus leading to support of H5. The results of testing H6 demonstrated that the effect of the interaction term (UF × Other-efficacy) on usage frequency (β = 0.12, *p* < 0.05) is positively significant at *p* < 0.05. That is, other-efficacy significantly moderates the effect of use innovativeness on both usage variety and usage frequency.

We examined the effect size of each predictor using the range of effect size defined by Cohen [49]. The effect size is calculated as the increase in *R*^2^ relative to the proportion of variance in the endogenous latent variable that remains unexplained, and, accordingly, it is divided into large (*f*^2^ = 0.35), medium (*f*^2^ = 0.15), and small (*f*^2^ = 0.02) effects. As shown in Table 3, the effect size (*f*^2^) ranges from 0.03 to 0.11. The goodness of fit of the entire structural model is 0.26, achieving a level of acceptance.

## 5. Conclusions

The prevailing focus of consumer innovativeness on adoption behavior fails to recognize the consumer post-adoption usage behavior such as usage patterns, a vital variable in explaining behavioral outcomes like revisit intention. Moreover, with the increasing attention to other-efficacy of consumer innovativeness research, explorations for its impacts have become more critical. Particularly, the moderating role of other-efficacy is largely unknown and uninvestigated. Our study complements extant research by specifying how use innovativeness influences usage behavior, and ascertaining the effects of other-efficacy on fitness customers’ usage patterns in a highly facility-driven services setting.

### 5.1. Theoretical Implications

The empirical results confirmed that use innovativeness is positively related to both usage variety and usage frequency, which is in agreement with prior findings of hand calculator [7], multi-functional products [8], and IT services [9]. The fitness center provides diverse fitness equipment for customers’ choices and adoption. Between the two types of usage pattern, variety of use assumes a slightly more central position because it is one of the key elements of use innovativeness [7]. Intuitive novelty-seeking prompts the innovative customers to enjoy various workout experiences and to explore different practices of the equipment, which thereby contributes to the extent of usage variety. Meanwhile, innovative fitness customers may incline to gain hedonic achievement during their workout, which could prompt them to spend more time enjoying the equipment.

Among the findings that should be highlighted is the moderating role of other-efficacy. The results correspond to the argument that other-efficacy indeed explains unique variance in customers’ participation and behavior [7]. In the context of fitness centers, innovative fitness customers tend to enjoy the workout routines more and use various machines frequently when they believe their partners or instructors have a higher level of efficacy to assist them. Fitness customers expect professional guidance and advice from their partners or trainers—so the perceptions of their partner’s efficacy are critical in determining the usage behavior. Furthermore, the customers’ perceived efficacy of their partners or trainers could also bolster their own self-efficacy to effectively operate the fitness equipment, which highlights the importance of assessing the other-efficacy belief.

This study made two clear contributions to the existing consumer innovativeness research. First, the primary contribution of this study was to verify the moderating role of other-efficacy between usage patterns and consumer attitude towards continuing the relationship with a fitness firm under the effect of use innovativeness. That is, we examined how other-efficacy strengthens the relationships between an innovative fitness consumer’s usage patterns and revisit intention. Second, usage variety and frequency increases fitness customers’ revisit intention. This outcome is congruent with prior studies [9,33], which found that customers with a greater tendency for usage variety and usage frequency are more inclined to be pleased with a service and to repurchase it again in the future. As well, the positive effect of usage rate is similar to Huh and Kim’s [28] outcome of cell phone purchases, which showed that high usage rate may result in product upgrade behavior on the successive versions of innovations.

When a workout environment provides low stimulation, fitness customers may feel bored and the desire for increased stimulation will rise simultaneously. This seems to lead customers to deep exploration, novelty seeking, or variety seeking for reaching their optimal level of stimulation [36]. Moreover, other-efficacy in fitness partners or trainers performs a supplementary role. Fitness customers that train together may coach each other in various ways of using fitness equipment or share workout experiences through cooperative or even competitive interaction. Thus, customers that exercise with partners go through co-creation experiences as they learn from each other to improve their skills and ways of using varied equipment. Likewise, trainers may not only co-develop with customers a series of workout programs, such as weight loss programs, but assist them to achieve certain training goals. This process of co-creation seemingly motivates the fitness customers to revisit the center for continuing the training.

### 5.2. Managerial Implications

Marketers of fitness centers should develop appropriate marketing strategies and improve market performance by focusing on the needs of different segments. We propose a fourfold segment of fitness customers using two constructs for managerial applications: use innovativeness (high and low) and other-efficacy (high and low).

#### 5.2.1. The Segment of High Use Innovativeness and High Other-Efficacy (*n*= 29)

The customers of this segment possess the characteristics of high use innovativeness and high other-efficacy. They are best described as pioneers of new fitness equipment because they place heavy reliance on their partners or trainers for innovative learnings. They are equipment heavy users as well. Adopting Pine and Gilmore’s [18] suggestion, we urge that fitness service providers should regularly refresh the consumer experiences, reintroduce service innovations, and renew its physical equipment. Regular innovation, in both service programs and the physical equipment, is an effective strategy to improve market performance and retain the service firm’s positioning. Updated equipment signals high levels of commitment to this segment of customers. Moreover, fitness centers should consider offering promotion programs such as buddy or group-together coupons. These incentives will encourage the fitness customers to invite and co-exercise with their workout partners, which in turn should increase the flow of fitness goers.

#### 5.2.2. The Segment of High Use Innovativeness and Low Other-Efficacy (*n* = 73)

The customers of this segment are low other-efficacy users, but they are interested in fitness workouts using different kinds of equipment. They have an innate curiosity and confidence of trying different workout machines without the assistance and/or shared experiences with peers or an instructor. Focus on educating this segment with knowledge on how to use existing and new machines. Furthermore, fitness firms should urge this segment of customers to work out with friends who are novices, because these innovative users could act as a facilitator for the fitness beginners. For this segment, marketers of fitness centers should design some goal-oriented programs, such as 5 k weight loss per person within a month. Through these co-practicing programs, these customers will likely share innovative usage experiences with others.

#### 5.2.3. The Segment of Low Use Innovativeness and High Other-Efficacy (*n* = 65)

The fitness customers in this segment have limited equipment usage patterns because they are unwilling or unable to use equipment in a novel way. Assistance and instruction from partners or instructors play a critical role for them because they feel inadequate to achieve desired outcomes. Professional instruction on how to use functional devices should encourage them to augment their usage patterns. Meanwhile, the trainer could boost these customers’ self-efficacy through positive verbal persuasion (e.g., “You did a great job”), because self-appraisals often form in response to the evaluative reactions from significant others, particularly in more complex, interpersonal contexts in which social cues are salient indicators of performance [19]. Additionally, verbal persuasion from workout partners or trainers may also encourage these customers to use fitness equipment in new ways. Thus, fitness management could offer a plus-one coupon or discount, inviting co-exercising practices.

#### 5.2.4. The Segment of Low Use Innovativeness and Low Other-Efficacy (*n* = 38)

This segment of customers has neither curiosity in using equipment nor confidence in their partners or trainers. Lacking intrinsic motivation to practice unfamiliar equipment, they need more help in instruction and coaching than other customers. Professional coaching and personal care would be crucial for their continual re-patronage. The fitness center could intentionally convey positive, realistic efficacy-related messages about their trainers to them, such as by displaying certificates or performance awards or providing a third party’s (e.g., supervisors) positive comments or praises to enhance their perception of other-efficacy from the instructor [50]. Additionally, the fitness club marketer may offer short-term coaching coupons and incentives for the customers to practice with different fitness equipment in a designated period. Furthermore, the marketer may invite the users of high use innovativeness and other-efficacy to help encourage the customers to use fitness equipment for various muscle trainings. Doing this should not only arouse their interests, but lay a foundation for highly innovative users to share their personal workout experiences.

### 5.3. Limitations and Future Research

Several limitations should be considered when interpreting the results and designing further research. First, this study examines use innovativeness, usage patterns, and the moderating role of other-efficacy in a particular type of close relationship (i.e., fitness customer–workout partner/trainer dyads); further research should explore similarities and differences in results across other facility-driven service contexts, such as self-service technologies. Contextual differences would offer an opportunity to identify some of the boundary conditions of our proposed framework and hypotheses. Second, this research did not take customers’ emotional responses into consideration. Experiential or hedonic values, such as enjoyment and excitement, are likely to play an important role in determining usage rates within varying service contexts. Third, other-efficacy is perceptual (also referred to as first-order expectations), and arguably has direct effects in close relationships together with self-efficacy. Fourth, the low R-squared values obtained in our study can be attributed to the theoretical model’s initial reliance on a single predictor. To enhance the predictive power of future research on this topic, we recommend that researchers consider a broader research framework that incorporates additional predictive constructs, such as domain-specific innovativeness (DOI). By incorporating multiple predictors, such research frameworks could offer greater explanatory power and enable more accurate predictions.

Further research should investigate the role of meta-perceptions (also referred to as second-order expectations), such as relation-inferred self-efficacy (RISE) and estimations of the other person’s self-efficacy (EOSE) beliefs, which are second-order efficacy perceptions regarding the expectations that a significant other person holds for oneself, and ascertains their relationship to the other-efficacy to contribute to a fuller understanding of their nature within dyadic contexts. The examination of the interaction effects among these three types of efficacy beliefs may provide facility-driven service marketers with further managerial insights.

## Figures and Tables

**Table 1:** Means, standard deviations, and standardized loadings of manifest variables.

Construct	Indicators	Mean	S.D.	Loading
Use Innovativeness (UI)	UI1	5.37	1.11	0.68 ***
	UI2	4.98	1.18	0.79 ***
	UI3	4.57	1.22	0.79 ***
Other-efficacy (OE)	OE1	5.23	1.05	0.92 ***
	OE2	5.28	1.05	0.95 ***
	OE3	5.18	1.11	0.96 ***
	OE4	5.25	1.10	0.93 ***
Usage Variety (UV)	UV	6.28	2.89	n.a.
Usage Frequency (UF)	UF	3.54	2.34	n.a.
Revisit Intention (RI)	RI1	5.66	0.84	0.91 ***
	RI2	5.44	0.95	0.91 ***
	RI3	5.68	0.88	0.92 ***

*** denote significance at the 0.001 level (two-tailed test), *n* = 205, n. a. = not applicable.

**Table 2:** Convergent validity, discriminant validity, and reliability of measurement model.

	Means (SD)	Cronbach’s *α*	CR	AVE	UF	OE	RI	UI	UV
UF	3.54(2.34)	n.a.	*n.a.*	*n.a.*	*n.a.*				
OE	5.24(1.01)	0.96	0.97	0.88	0.20	*0.94*			
RI	5.59(0.81)	0.90	0.94	0.83	0.27	0.35	*0.91*		
UI	4.97(0.89)	0.63	0.80	0.57	0.31	0.26	0.47	*0.75*	
UV	6.28(2.89)	n.a.	*n.a.*	*n.a.*	0.35	0.20	0.27	0.26	n.a.

The italicized values denote the square root of the average variance extracted (AVE), n.a. = not available because of a single measuring item. UF = Usage Frequency, OE = Other-efficacy, RI = Revisit Intention, UI = Use Innovativeness, UV = Usage Variety, CR = Composite Reliability.

**Table 3:** Structural model results and effect sizes (*f*^2^).

Criterion	Predicators	*R* ^2^	Path Coefficient	*f* ^2^
Use Variety	Use Innovativeness	0.11	0.19 (t = 2.79) **	0.08
Usage Frequency		0.12	0.24 (t = 4.66) ***	0.04
Revisit Intention	Usage Variety (UV)	0.11	0.20 (t = 2.99) ***	0.11
	UV × Other-efficacy		0.16 (t = 2.90) **	0.03
Revisit Intention	Usage Frequency (UF)	0.11	0.20 (t = 2.96) **	0.04
	UF × Other-efficacy		0.12 (t = 2.10) *	0.04

* Significant at <0.05 level; ** significant at < 0.01 level; *** significant at < 0.001 level; effect size measures the relevance of each predictor of a dependent latent variable, and is based on the relationship of determination coefficients when including or excluding a particular predictor from the structural equation; GoF (Goodness of Fit) = 0.26.
